# Exploring the Kinetics and Thermodynamics of a Novel Histidine Ammonia-Lyase from *Geobacillus kaustophilus*

**DOI:** 10.3390/ijms251810163

**Published:** 2024-09-21

**Authors:** Francisco Manuel Salas-Garrucho, Alba Carrillo-Moreno, Lellys M. Contreras, Felipe Rodríguez-Vico, Josefa María Clemente-Jiménez, Francisco Javier Las Heras-Vázquez

**Affiliations:** 1 Departamento de Química y Física, Universidad de Almería, 04120 Almería, Spain; fransg@ual.es (F.M.S.-G.); albacarrillo@ual.es (A.C.-M.); fvico@ual.es (F.R.-V.); jmclemen@ual.es (J.M.C.-J.); 2 Campus de Excelencia Internacional Agroalimentario ceiA3, Universidad de Almería, 04120 Almería, Spain

**Keywords:** histidine ammonia-lyase, enzymatic kinetics, thermostability, thermal inactivation, circular dichroism

## Abstract

Histidine ammonia-lyase (HAL) plays a pivotal role in the non-oxidative deamination of L-histidine to produce *trans*-urocanic, a crucial process in amino acid metabolism. This study examines the cloning, purification, and biochemical characterization of a novel HAL from *Geobacillus kaustophilus* (*Gk*HAL) and eight active site mutants to assess their effects on substrate binding, catalysis, thermostability, and secondary structure. The *Gk*HAL enzyme was successfully overexpressed and purified to homogeneity. Its primary sequence displayed 40.7% to 43.7% similarity with other known HALs and shared the same oligomeric structure in solution. Kinetic assays showed that *Gk*HAL has optimal activity at 85 °C and pH 8.5, with high thermal stability even after preincubation at high temperatures. Mutations at Y52, H82, N194, and E411 resulted in a complete loss of catalytic activity, underscoring their essential role in enzyme function, while mutations at residues Q274, R280, and F325 did not abolish activity but did reduce catalytic efficiency. Notably, mutants R280K and F325Y displayed novel activity with L-histidinamide, expanding the substrate specificity of HAL enzymes. Circular dichroism (CD) analysis showed minor secondary structure changes in the mutants but no significant effect on global *Gk*HAL folding. These findings suggest that *Gk*HAL could be a promising candidate for potential biotechnological applications.

## 1. Introduction

The ammonia-lyase family encompasses a group of homologous enzymes in the degradation of amino acids via deamination. In contrast to the usual oxidative deamination catabolic route, ammonia-lyase enzymes carry out a non-oxidative deamination of the α-amino group via elimination of a non-acidic β hydrogen, giving the correspondent acids [1,2]. Within the family, some of the most representative and studied enzymes are phenylalanine ammonia-lyases (PALs; EC 4.3.1.5), histidine ammonia-lyases (HALs; EC 4.3.1.3), aspartate ammonia-lyases (AALs; EC 4.3.1.2), and tyrosine ammonia-lyases (TALs; EC 4.3.1.23) [3,4]. Over the years, it was thought that this group was characterized by a catalytical essential dehydroalanine site [5,6,7]. However, more recent studies demonstrate a common characteristic active site containing 3.5-dihydro-5-methylene-4H-imidazole-4-one, known as the MIO catalytic cofactor [8,9,10]. This highly electrophilic moiety is formed post-translationally and autocatalytically from the Ala-Ser-Gly sequence [2,4].

The enzymes characterized in this study are the wild-type HAL and three mutant variants. HAL, considered a universal enzyme, is supposed to be the first enzyme and principal regulator of the non-oxidative degradation pathway of L-histidine amino acid [11,12]. HAL carries the degradation of L-histidine to form the α,β-unsaturated *trans*-urocanate and releases ammonia [3,4,8,12]. The reaction mechanism is proposed to be following a Friedel-Crafts attack [2,3,13,14]. In this mechanism, the electron pair from the imidazole of (S)-His attacks MIO, leading to the formation of a cation on this imidazole ring, which in turn acidifies the hydrogen atom at the β-position of (S)-His. Afterwards, the enzyme’s basic group abstracts the β-proton, resulting in the formation of urocanate and the elimination of ammonia, thereby regenerating the electrophilic group. Other analogous enzyme mechanisms have been extensively studied. For example, PAL and AAL demonstrate a comparable non-oxidative reaction involving their respective substrates, leading to the formation of trans unsaturated carboxylic acid through a shared Friedel–Crafts mechanism [10,13,15,16]. Regarding the substrate specificity of aromatic ammonia-lyases, it has been demonstrated that it is limited to the individual aromatic amino acids: L-histidine, L-phenylalanine, and L-tyrosine. Despite a single, unconfirmed report of a tryptophan ammonia-lyase [17], naturally occurring MIO-dependent enzymes with alternative specificities remain undiscovered.

Even though HAL shares the same catalytic moiety as other enzymes of the family (PAL, TAL, and AAL), it is only active within a narrow substrate range, specifically 2 and 4-fluoro-L-histidine [18], 4-nitro-L-histidine [19], L-histidine methyl ester [20], L-histidine [13,19,20], and Nτ-methylhistidine [21]. It was observed that zinc increases the activity of HAL from *Pseudomonas putida* (*Ppu*HAL) during L-histidine degradation [20,22]. Such metal activation could be explained by assuming its interaction with H83 and the substrate histidine [23]. However, in the X-ray crystal structure of *Ppu*HAL, no Zn^2+^ ion is observed [24].

HAL is fairly ubiquitous in nature, being present in a wide range of organisms, from bacteria to humans, spanning through plant and animal species. HAL has been isolated from several biological sources, and the sequence and functional overexpression of the gene *hutH* have been reported [9,20,24,25,26,27,28,29]. The crystal and active site structures of these enzymes have been previously studied [24,25,26,27,28,29]. Based on the eight reported crystal structures, the HAL structures are homotetramers with four identical active sites formed by amino acids from three distinct monomers.

Histidine is one of the nine essential amino acids crucial for the metabolism of most animals, necessitating its acquisition through the diet. In mammals, a lack or defect of HAL can lead to diseases such as histidinemia, a genetic metabolic disorder characterized by elevated levels of the amino acid histidine in the human body, and low levels of urocanic acid, which serves as a UV protector in the skin [30]. However, in most bacteria, it only blocks the histidine utilization route, which is not lethal. The pathway of histidine catabolism is highly conserved among bacteria, enabling cells to utilize this amino acid as a source of carbon, energy, and nitrogen [12]. HAL enzymes have been extensively studied in several bacterial genera, including *Pseudomonas*, *Bacillus*, *Serratia*, and *Achromobacter*, among others [3,8,12,13,24,25,26,27], as well as in some extremophiles [29]. HALs have promising industrial applications, such as their reported role in producing optically pure D-histidine [31]. More recently, it has been shown that bacterial HALs possess an antineoplastic effect and can selectively inhibit cell proliferation, highlighting their potential for therapeutic use [32,33,34]. Additionally, HAL from *Kurthia* sp. (*Kx*HAL) has been used to produce *cis*-urocanic acid, a stereoisomer with immunosuppressant properties. Moreover, in vitro studies indicate that *Kx*HAL can reduce viral proliferation [34].

In our endeavor to obtain a new HAL with superior robustness for industrial applications compared to those previously described, our laboratory began screening thermophilic microorganisms. In this context, thermostable enzymes derived from thermophilic and extremophilic microorganisms emerge as appealing options for industrial processes operating in harsh conditions [35]. Thermozymes have been demonstrated to offer numerous advantages, such as increased tolerance and high reaction rates at elevated temperatures. The genus *Geobacillus* mainly comprises Gram-positive thermophilic bacteria. The NCBI taxonomy browser currently lists 182 complete genome sequences for *Geobacillus* species (https://www.ncbi.nlm.nih.gov/Taxonomy/Browser/wwwtax.cgi (accessed on 30 August 2024)). One of these is the genome of *Geobacillus kaustophilus*, a bacterium with a maximum growth temperature limit of 74 °C and an optimal growth temperature of 60 °C. The genome of *G. kaustophilus* consists of a chromosome of 3.54 Mb and a single plasmid of 47.9 kb [36]. In their study, the authors assigned biological roles to 1914 CDSs, one of which corresponds to a putative HAL. Therefore, the capability of *G. kaustophilus* to thrive in harsh conditions makes it a valuable source of thermostable enzymes.

In this work, we describe the cloning, purification, and biochemical characterization of a *Gk*HAL enzyme, along with eight other active site mutants. These HAL mutants were produced to evaluate their importance in substrate binding and catalysis through enzyme kinetic behavior. We tested enzyme activity with various substrates and discovered that L-histidinamide is a new substrate for two out of the eight HAL mutants. The kinetic and thermal characteristics of both the wild-type *Gk*HAL and its mutants are also examined and discussed. Our results show that *Gk*HAL exhibits kinetic thermostability, achieving maximum activity at 85 °C. Kinetic and thermodynamic studies allowed us to test enzyme efficiency with each substrate. Circular dichroism (CD) spectra and thermal denaturation were performed to understand the influence of such mutations on the secondary structure composition of *Gk*HAL and its thermostability features.

## 2. Results

### 2.1. Sequence Analysis of Histidine Ammonia-Lyase from Geobacillus kaustophilus CECT4264

**Figure 1:**
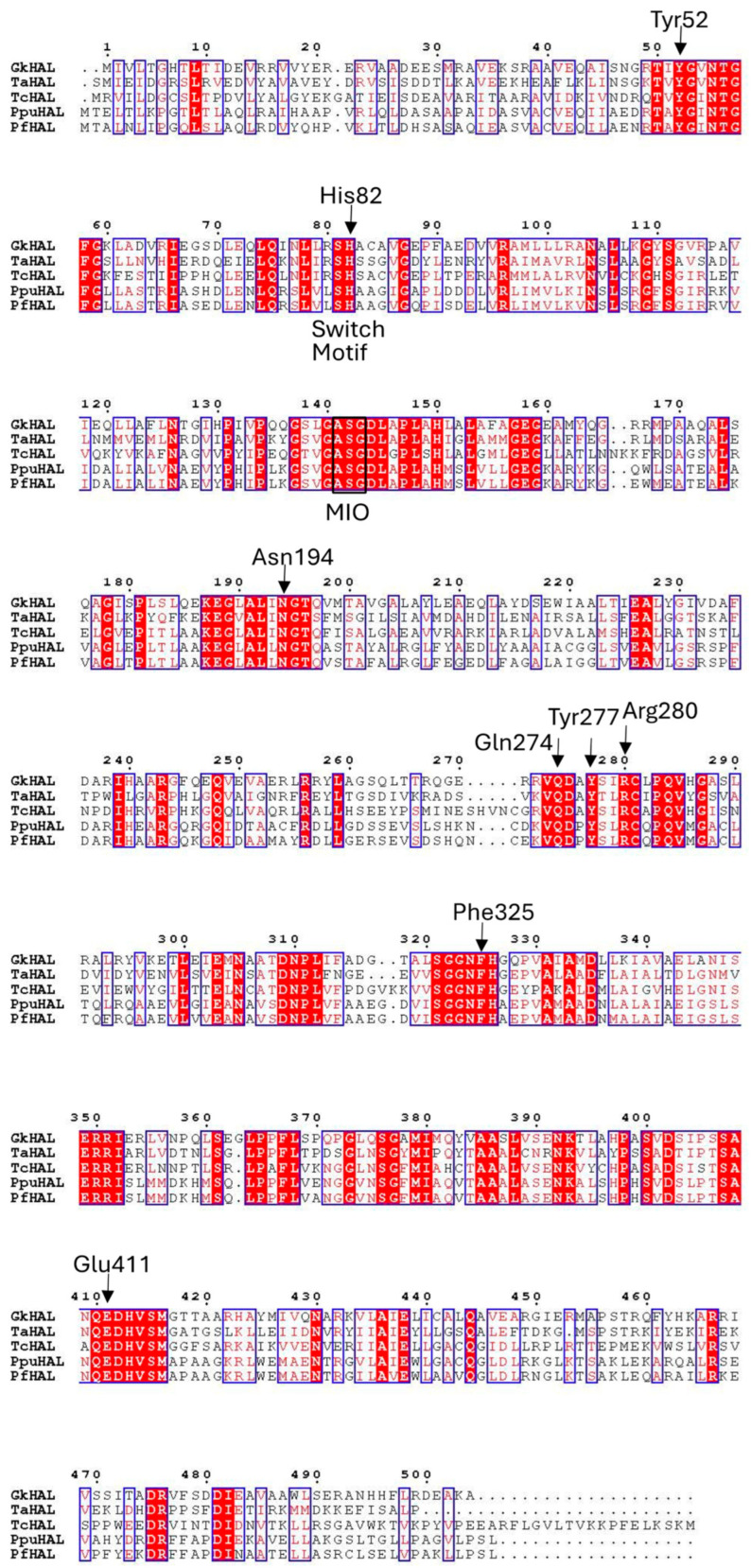
Sequence alignment for histidine ammonia-lyases of contrasted activity. GkHAL, *Geobacillus kaustophilus* CECT4264, Genbank acc. No. PQ065632; TaHAL, *Thermoplasma acidophilum*, Uniprot Q9HLI6; TcHAL, *Trypanosoma cruzi*, Uniprot Q4E133; PpuHAL, *Pseudomonas putida*, Uniprot P21310; PfHAL, *Pseudomonas fluorescens*, NCBI Reference Sequence No. WP_003220818.1.

*G. kaustophilus* CECT4264 showed a distinct fragment for the *hutH* gene (*gkhutH*). The GenBank accession number of *Gk*hutH is BAD74670.1. The gene encompassing the region 430274–431788 encodes a polypeptide of 504 amino acids with a calculated molecular mass of 54.7 kDa. The amino acid sequence deduced for *Gk*HAL was compared to HAL sequences with demonstrated activity or solved structures (Figure 1). The highest similarity in amino acid sequence (43.7%) was found with HAL from *Thermoplasma acidophilum* [29], whereas the lowest identity percentage (40.7%) was observed when comparing the *G. kaustophilus* sequence with HAL from *Trypanosoma cruzi* [28]. When contrasting with sequences from the *Pseudomonas* genus, the identity regarding *P. putida* (*Ppu*) was 42.3% [24], whereas in comparison with *P. fluorescens* (*Pf*), the identity decreased to 41.9% [27]. All of these HAL sequences feature a conserved amino acid triad, Ala-Ser-Gly, capable of forming the 3,5-dihydro-5-methylidine-4H-imidazol-4-one (MIO) prosthetic group, which is found in most of the aromatic ammonia-lyases identified to date [8,37]. The *Gk*HAL sequence also reveals the presence of the characteristic substrate switch motif, Ser/His, for ammonia-lyase activity [27], identified at positions 81–82. Furthermore, as depicted in Figure 1, this dyad is indeed present within the aromatic binding region of the other HALs. Additionally, the multiple alignments showed that *Gk*HAL contains conserved catalytic active sites, which are similar to those found in other homologous HAL proteins. For instance, the amino acid residues Y and E, which are predicted to coordinate the carboxylic and amino groups of the substrate L-histidine, were conserved in *Gk*HAL (Tyr52 and Glu411). Moreover, the amino acid residues N, Q, and R, which participate in hydrogen bonding to the ammonium function of the substrate [25] and in anchoring the carboxylate moiety of the substrate or the cationic intermediate, are highly conserved in *Gk*HAL (Asn194, Gln274, and Arg280). On the other hand, the active site residue Y277 in *Gk*HAL is possibly involved in assisting Glu411 in its key catalytic role, while the residue Phe325 may stabilize the cationic intermediate, as shown previously in *Ppu*HAL [25]. These findings suggest that the *gkhutH* gene encodes a functional HAL protein.

### 2.2. Expression, Production, and Purification of GkHAL and Its Active Site Mutants

**Figure 2:**
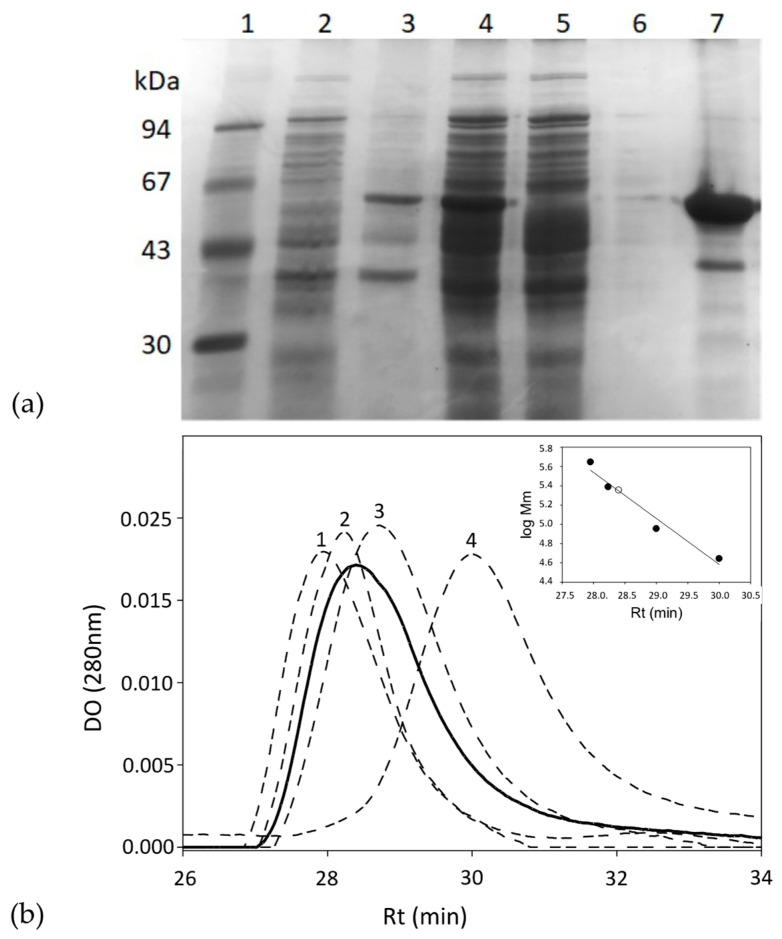
(**a**) SDS-PAGE analysis of each purification step of the *Gk*HAL wild-type from *E. coli* BL21 harboring the pJMC93 plasmid. Lane 1: low molecular weight marker; lane 2: non-induced precipitate; lanes 3 and 4: pellet and supernatant of the resuspended crude extract after cell sonication; lane 5: flow-through after adding the sonicated supernatant to the metal affinity column; lane 6: eluate after washing the metal affinity column with buffer; lane 7: purified enzyme. (**b**) Size-exclusion chromatography of the purified *Gk*HAL wild-type (black line) and molecular weight markers (dashed line). The molecular weight markers consisted of (1) apoferritin from equine spleen (443 kDa), (2) dihydropyrimidinase from *Sinorhizobium meliloti* CECT4114 (*Smel*Dhp) (245 kDa) [39], (3) L-carbamoylase from *Geobacillus stearothermophilus* CECT43 (*Bs*Lcar) (44 kDa) [40], and (4) chicken ovalbumin (44 kDa). The inset represents the fit of the retention time (Rt) versus the logarithm of the molecular masses (Mm) of the protein standards (black circles) and that obtained for *Gk*HAL (white circle).

The *gkhutH* gene was inserted into the pDANI3 expression vector with *Nde*I and *Bam*HI restriction enzymes to produce the C-terminal His6-tagged *Gk*HAL. The *Gk*HAL enzymes were functionally expressed in *E. coli* BL21 and purified in one step using immobilized cobalt affinity by chromatography [38]. SDS-PAGE analysis of the purified wild-type *Gk*HAL enzyme revealed a band with 95% purity and an apparent molecular mass of approximately 55 kDa (Figure 2a), which is consistent with the deduced molecular mass from the amino acid sequence (56.5 kDa). The protein *Gk*HAL was eluted at pH 8.5 at 28.4 min in the Biosep-SEC-S2000 column (HPLC), which yields an apparent molecular mass of 220 kDa (Figure 2b), suggesting a tetrameric structure for the protein in solution.

The purity and monomeric molecular weight of the purified mutated enzymes were also analyzed using SDS-PAGE gel electrophoresis (Figures S1–S7). Enzymes were obtained at yields ranging from 26 to 74 mg of pure protein per liter of cell culture. In addition, among the mutated enzymes analyzed, *Gk*HAL Y277F was the only one that could not be expressed or purified, suggesting that this residue has an essential role in conformational stability.

### 2.3. Protein Characterization of GkHAL and Its Mutants Followed by Activity Measurements

The activities of both the *Gk*HAL wild-type and its mutants were compared by measuring their ability to convert histidine into *trans*-urocanic acid under standard assay conditions. Mutations at residues Y52F, H82L, N194D, and E411D resulted in enzyme forms that failed to produce *trans*-urocanic acid, indicating that these mutations hindered the enzymes’ capability to catalyze the reaction. The Q274N, R280K, and F325Y mutated enzymes were able to produce the reaction but with less activity compared to the *Gk*HAL wild-type. Mutants with analogous changes in these residues in *Ppu*HAL also showed a lesser reduction in activity loss [25], pointing to the conservative nature of the active site in histidine ammonia-lyases.

We investigated the effect of pH (2–12) on the activity of *Gk*HAL and its mutants (Q274N, R280K, and F325Y) at 65 °C using L-histidine as the substrate. The pH range of the purified proteins is illustrated in Figure S8. All proteins displayed maximum activity between pH 7.5 and 8.5, with activity declining below pH 7 and above pH 9. The maximum and optimum pH of 8.5 for *Gk*HAL enzymes align with data from HALs of other bacterial strains such as *P. putida* [20], *P. fluorescens* R124 [27], and *T. acidophilum* [29].

The effect of temperature on enzyme activity of *Gk*HAL and its mutants (Q274N, R280K, and F325Y) was assayed at different temperatures ranging from 20 to 98 °C, at pH 8.5. Both the wild-type enzyme and the Q274N and R280K mutants showed maximum activity at 85 °C, while the F325Y mutant exhibited maximum activity at 80 °C (Figure S9). As thermal stability studies on the activity of wild-type *Gk*HAL revealed a gradual loss of activity after preincubation for 15 min at temperatures above 70 °C (Figure S10), the reactions were conducted at 65 °C.

To explore the substrate specificity of the enzymes, their activity was tested with various substrates. We began by measuring the activity of the other proteinogenic amino acids as possible substrates, but none of them were substrates for *Gk*HAL or its mutants, confirming its stricter substrate specificity [9,13,19,27]. Among the L-histidine homologous substrates tested, all proteins showed activity with L-histidine methyl ester. However, this behavior differs because the highest rate of urocanate methyl ester formation was detected for the wild-type *Gk*HAL and the mutant Q274N, whereas the mutants R280K and F325Y exhibited a slow activity rate under identical assay conditions (Table 1). Previous investigations have shown that L-histidine methyl ester can serve either as a substrate [20] or as a competitive inhibitor [41]. Furthermore, the mutated enzymes R280K and F325Y exhibited activity with L-histidinamide (Table 1). However, the mutant R280K showed a 13-fold higher rate of urocanamide production compared to F325Y. Neither the wild-type nor the Q274N-mutated *Gk*HAL catalyzed the reaction with L-histidinamide.

### 2.4. Kinetic and Thermal Characterization of Enzymes

Kinetic parameters of *Gk*HAL and its mutants were measured at varying substrate concentrations between 0.25 and 50 mM. *K*_m_ values were calculated for L-histidine and L-histidine methyl ester for all enzymes and for L-histidinamide for those that showed to carry the reaction. Maximum velocity *V*_max_, *K*_m_, *k*_cat_, and catalytic efficiency were obtained for each protein and substrate combination (Table 1).

A *K*_m_ value comparison indicates that all mutants have similar affinities for L-histidine as a natural substrate. However, the *Gk*HAL mutant Q274N showed a higher *K*_m_ value (12.58 mM) than the other enzymes (3.38–7.99 mM). Using L-histidine methyl ester as a substrate resulted in a similar trend, with higher *K*_m_ values observed for the mutants Q274N and F325Y. Considering that the R280K mutation resulted in a low *K*_m_ (3.43 mM), it can be inferred that this mutant exhibits a higher binding affinity with L-histidine methyl ester compared to L-histidine itself. On the other hand, mutant enzymes capable of catalyzing the reaction with L-histidinamide displayed comparable *K*_m_ values to those of L-histidine, reflecting similar affinities for both substrates.

Although *Gk*HAL mutants exhibited similar *K*_m_ values when L-histidine was used as a substrate, their *k*_cat_ values were 6 to 127 times lower than that of wild-type *Gk*HAL, indicating that they are less active. The *k*_cat_ values determined for L-histidine methyl ester followed the same trend as those observed for L-histidine, suggesting that the mutant enzymes react with it at a significantly lower rate. The effect of the R280K mutation is somewhat controversial, as it shows a low *K*_m_ with L-histidine methyl ester, yet the resulting *k*_cat_ is low (0.090 s^−1^), indicating strong binding between the enzyme and substrate but poor catalytic function.

Catalytic efficiency is defined as the relationship between catalytic function and substrate binding, represented by the ratio *k*_cat_/*K*_m_. As expected, the wild-type *Gk*HAL exhibited the highest efficiency with its standard substrate, L-histidine (2.5367 mM^−1^ s^−1^). Furthermore, the catalytic efficiency values were also highest with the wild-type enzyme for L-histidine methyl ester. In contrast, mutated enzymes show 16 to 211 times less efficiency. In relation to L-histidinamide, the R280K mutant demonstrated the highest catalytic efficiency. However, this efficiency is 62 times lower than that of the wild-type enzyme with L-histidine.

**Figure 3:**
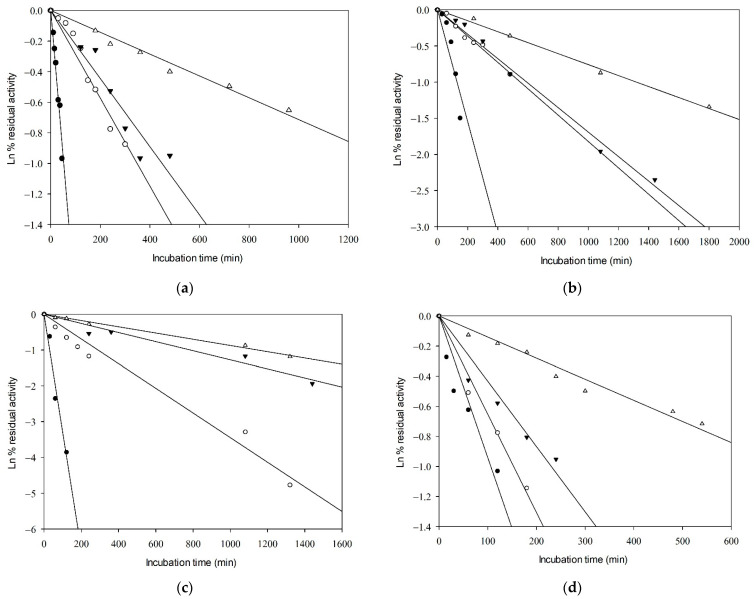
Kinetics of thermal inactivation of the catalytic activity of (**a**) wild-type *Gk*HAL, (**b**) Q274N *Gk*HAL, (**c**), R280K *Gk*HAL, and (**d**) F325Y *Gk*HAL. Incubation temperatures shown are 80 °C (close circles), 75 °C (open circles), 70 °C (close inverted triangles), and 65 °C (open triangles).

The thermal inactivation parameters of *Gk*HAL proteins were studied at temperatures of 65, 70, 75, and 80 °C in 100 mM DEA buffer (pH 8.5) (Table 2). The exponential decay was analyzed using semi-logarithmic plots. Linear Arrhenius plots were obtained, indicating first-order inactivation of the enzymes in all cases (Figure 3). Steeper slopes indicate faster inactivation rates. As expected, higher temperatures resulted in shorter times for enzyme inactivation. The inactivation rate constant (*k*_d_) was derived from the slopes of the Arrhenius plots, with higher values indicating more rapid inactivation. The half-life times (t_1/2_) of the active enzymes were determined from the inactivation rates. The thermal inactivation parameters, including free energy (ΔG*), enthalpy (ΔH*), and entropy (ΔS*) of the active enzymes (Table 2), were calculated as described in references [42,43].

As depicted in Table 2, an increase in temperature led to a decrease in t_1/2_ values and an increase in the first-order heat inactivation rate constants (*k*_d_) for both the *Gk*HAL wild-type and its mutants. At 65 °C, the wild-type *Gk*HAL exhibited the highest half-life, indicating greater stability compared to the mutated enzymes. At 80 °C, the trend reversed, with the wild-type enzyme showing a shorter half-life compared to the mutated enzymes, except for the R280K mutation. Therefore, mutated enzymes appear to exhibit better stability at high temperatures. The activation energy for deactivation (E_a_) represents the amount of energy molecules need to absorb to begin enzyme inactivation and attain the height of the barrier, or transition state, for inactivation. Regarding E_a_, the Q274N, R280K, and F325Y mutations exhibited lower values of 35.64, 44.41, and 25.11 kcal/mol, respectively, compared to the wild-type enzyme, which had 49.42 kcal/mol.

Thermodynamic parameters provide valuable insights into protein stability, revealing stabilization or destabilization effects that may not be evident from half-life times alone. The ΔG* value is directly related to protein stability; thus, a higher ΔG* indicates greater enzyme stability [44]. Free energy decreased with the rise in temperature in wild-type *Gk*HAL and mutants Q274N and R280K, suggesting the destabilization of the enzymes, which is consistent with the half-life and *k*_d_ results. This trend is also observed for the F325Y mutant in the temperature range of 338 to 348 K. The ΔG* values obtained for the different proteins were similar, indicating no significant stability differences. Table 2 also shows that ΔH* slightly decreases with rising temperatures in both *Gk*HAL and its mutated forms, indicating that less energy is required to denature the enzymes at higher temperatures. Additionally, the positive enthalpy values suggest that the inactivation process is endothermic [45].

Given that the activation energy for deactivation and the thermodynamic parameters of inactivation are typically higher for wild-type *Gk*HAL compared to the Q274N, R280K, and F325Y mutations, we can suggest that these mutants may be less stable against thermal inactivation.

### 2.5. Circular Dichroism of Enzymes

**Figure 4:**
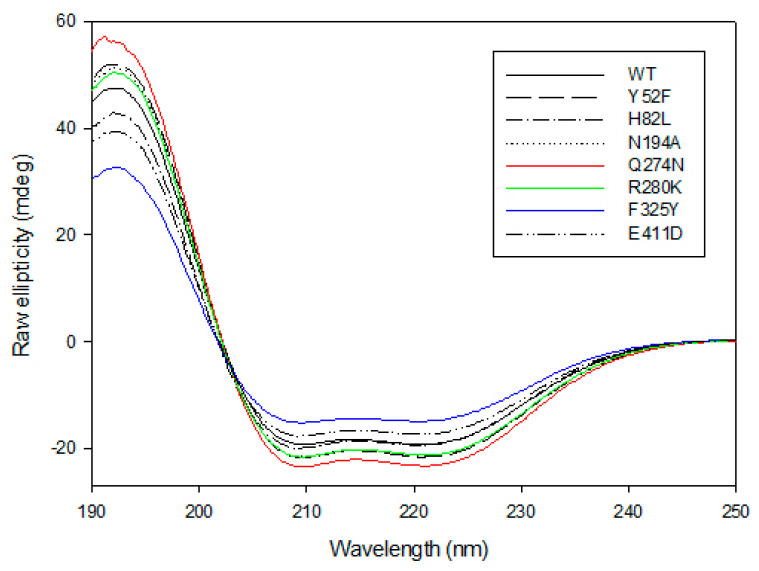
Far-UV CD spectra for *Gk*HAL enzymes recorded at 25 °C using a J-815 spectrophotometer. Six spectra were averaged for each curve.

The CD spectra of the wild-type *Gk*HAL and its mutants have been investigated experimentally (Figure 4). The CD spectrum of *GK*HAL at pH 8.5 exhibits two negative peaks at 210 and 222 nm and a positive peak at 192 nm. This is characteristic of a protein with an α-helical or turn-like conformation content, as well as a fraction of β-sheet structure [43], suggesting that the purified protein adopts a well-defined folded structure. The loss of ellipticity at 222 nm is attributed to α-helical content, while the loss at 210 nm indicates the presence of β-sheets in the structure of the protein. As shown in Figure 4, all mutants clearly show structural helicity, indicating that they keep their internal chirality. However, except for the H82L mutant, whose spectrum completely overlaps with that of *Gk*HAL, all other mutants show slight differences from the wild-type *Gk*HAL. Since the mutants Y52F, H82L, N194D, and E411D lacked activity on L-histidine, we did not analyze their CD spectra. To assess the slight variations in the secondary structures of mutants Q274N, R280K, and F325Y in comparison to the wild-type *Gk*HAL, we utilized the k2d algorithm available on DichroWeb [46]. This tool facilitated the determination of the percentages of α-helices, β-sheets, and random structures for each enzyme. Table 3 presents the deconvolution results, indicating the relative percentages of each secondary structure. Based on the CD data, the wild-type *Gk*HAL exhibits a conformation comprising 47% α-helix structure, 15% β-sheet structure, and 38% random coil elements. For the structure of the R280K mutant, the amount of α-helix structure is considerably higher, while the amount of β-sheet structure is distinctly lower compared to the wild-type enzyme. In contrast, the α-helical content in the Q274N and F325Y mutants decreases significantly, from 47% to 39% and from 47% to 35%, respectively, while the β-sheet content increases compared to the wild-type *Gk*HAL. Nonetheless, it is still difficult to ascertain whether these minor structural changes are connected to the newly observed activity in the R280K and F325Y mutants.

**Figure 5:**
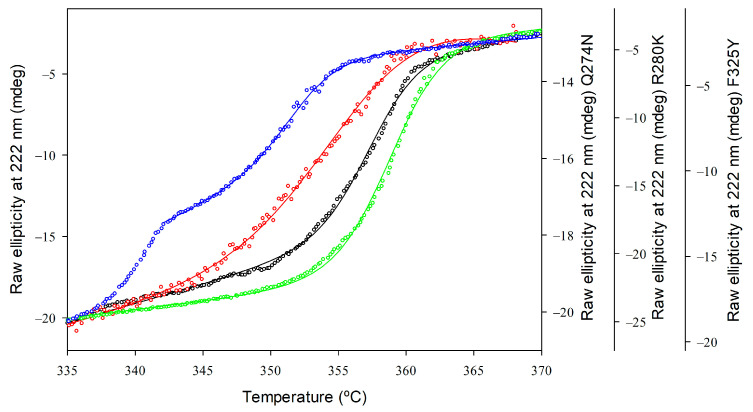
Thermal unfolding profiles of *Gk*HAL enzymes monitored by far-UV CD. Wild-type *Gk*HAL (black open circles) and *Gk*HAL mutants Q274N (red open squares), R280K (green open diamonds), and F325Y (blue open hexagons). All profiles were measured with a scan rate of 1 K/min.

The thermostability of *Gk*HAL variants was evaluated by observing temperature-induced unfolding using CD spectroscopy. Structural changes in protein folding were studied at a 4 µM protein concentration with a temperature interval of 62 and 95 °C, measuring the loss of ellipticity at 222 nm. Figure 5 presents the melting profiles of the variants, along with the associated non-linear least squares fit using the Gibbs–Helmholtz equation to determine the *T*_m_ values for each protein. For the wild-type *Gk*HAL, a single sigmoidal transition was observed with an apparent *T*_m_ (*T*_m(app)_) value of 357.36 ± 0.06 K, indicating that the thermal denaturation is a two-state process [47]. Compared to wild-type *Gk*HAL, the R280K mutant not only displays an increase in helical content but also shows a modest improvement in thermal stability, with a *T*_m(app)_ value of 358.80 ± 0.04 K. In contrast, mutants like Q274N and F325Y, which have reduced helical content, exhibited a melting transition with a decrease in apparent melting temperature by 2.16 K and 5.51 K, respectively, compared to the wild-type (Table 3).

We conducted a second CD spectrum to determine if the structure recovered after thermal denaturation. None of the proteins exhibited a spectrum similar to those in Figure 4. Furthermore, all the proteins showed aggregation after heating, suggesting that they unfolded into intermediates that were prone to aggregation. As a result, *Gk*HAL and its mutants cannot completely refold because partially unfolded species accumulate and aggregate, rendering the unfolding process irreversible and precluding thermodynamic analysis [48].

## 3. Discussion

### 3.1. Cloning, Expression, and Characterization of Histidine Ammonia-Lyase from Geobacillus kaustophilus

*G. kaustophilus,* a thermophilic bacterium capable of growing across a broad temperature range from 42 °C to a maximum of 74 °C, can serve as a valuable source for discovering highly thermoresistant enzymes [36,49,50]. In this study, we identified a putative *hutH* gene in *G. kaustophilus* HTA426 through genome analysis. To investigate the function of this gene product, the *hutH* gene was amplified from the genome of *G. kaustophilus* CECT4264 using PCR, enabling the construction of the recombinant plasmid pJMC93. The *Gk*HAL recombinant protein was then successfully expressed as a soluble protein, allowing us to further investigate its characteristics and functions.

Based on the multiple alignment of the deduced amino acid sequence of the cloned *Gk*HAL, we found more than 40% identity with the major HAL enzymes characterized to date [24,27,28,29]. Similar to other HAL enzymes, *Gk*HAL contains the amino acids that form the MIO internal cofactor, which acts as an electrophilic group essential for histidine deamination [4,8,37]. The recognition of histidine by HALs has been attributed to the presence of the SH motif in the aromatic binding region [51]. Other residues in this region may also be involved in substrate interaction, such as leucine at position 76 in *Ppu*HAL [52] and at position 80 in *Pf*HAL [27,52], alanine and serine at position 85 in *Ppu*HAL or *Tc*HAL [28], and methionine at position 380, which is conserved across all bacterial HALs described so far [28]. Given that these residues are fully conserved in *Gk*HAL, it is reasonable to infer a strict substrate specificity, aligning with previous reports for other HALs. Additionally, the sequence similarity reveals that *Gk*HAL shares the same active site residues as other HALs. In this context, the residues Tyr52 and Glu414, which are crucial for catalysis, are fully conserved. Furthermore, we identified Asn194, Gln274, and Arg280 in *Gk*HAL as equivalent residues that are thought to play crucial roles in substrate positioning and maintaining the active center [9,13,25]. The sum of the results indicates that the *hutH* gene of *Geobacillus kaustophilus* encodes a functional HAL protein. On the other hand, the similarity of the substrate binding and catalytic motifs of *Gk*HAL to those in other bacterial HALs suggests that it would exhibit similar catalytic properties.

Recombinant *Gk*HAL was successfully expressed and purified, enabling us to determine its enzyme activity and biochemical properties. It has been reported that *Ppu*HAL activity is stimulated by divalent metal ions such as Zn^2^⁺ [22], likely because the ion might coordinate the interaction between L-histidine and the imidazole group of His83, as previously suggested [23,25]. More recently, Csuka et al. reported that Zn^2^⁺ ions did not result in any further increase in the enzyme activity of *Pf*HAL [27]. In this context, we examined the effect of 10 μM Zn^2+^ on the reaction catalyzed by *Gk*HAL. The velocity ratio values of *Gk*HAL in the absence or presence of the metal ion were nearly identical (20.4 ± 1.4 nmol/min and 20.8 ± 0.9 nmol/min, respectively). Additionally, the enzyme activity was unaffected by 10 mM EDTA (21.0 ± 0.5 nmol/min). From the findings, it can be suggested that Zn^2+^ is not required for the enzyme activity of *G. kaustophilus* CECT4264 HAL. While Miranda et al. [28] measured *Tc*HAL activity in the presence of Mn^2^⁺, they did not address the role of these ions in the enzyme activity. Finally, there is no available information about the requirement for divalent metal ions in the most recently characterized HAL [29].

The monomeric size determined for the *Gk*HAL subunit is consistent with that observed in all recombinant histidine ammonia-lyase enzymes studied to date [20,21,27,28,29]. Our findings also suggest that the functional structure of *Gk*HAL is a tetramer. Notably, the crystal structures of *Ppu*HAL [24], *Tc*HAL [28], and *Ta*HAL [29] have revealed a homotetrameric organization with four active centers.

### 3.2. The Kinetics of GkHAL Enzymes: Comparison with Other HALs

Based on sequence analysis, Tyr52, His82, Asn194, Gln274, Tyr277, Arg280, Phe325, and Glu411 were identified as potentially important for catalysis, substrate positioning, and active center maintenance. These residues were therefore selected for site-directed mutagenesis to verify their functions. Comparisons of the activities of wild-type and mutant *Gk*HAL enzymes revealed new characteristics arising from these mutations. The *Gk*HAL mutations Y52F, H82L, N194D, and E411D showed no activity on L-histidine as the natural substrate. Thus, the amino acids Tyr52, His82, Asn194, and Glu411 must be essential for the active site structure and the formation of the enzyme-substrate complex. Changes in the composition of the specified sites led to the inactivation of the *Gk*HAL. Although similar mutants in *Pseudomonas putida* HAL showed activity on L-histidine, their *k*_cat_ values were 1,000 to 20,930 times lower than the wild-type enzyme [25]. On the other hand, the mutants Q274N, R280K, and F325Y were able to catalyze the elimination of ammonia from L-histidine, although at a lower rate than the wild-type *Gk*HAL enzyme. Comparison of the kinetic constants with those reported for similar mutants of *Ppu*HAL revealed that they exhibit the same trends, regardless of the reaction temperature (25 °C for *Ppu*HAL [25] and 65 °C for *Gk*HAL (this work)). Thus, it can be concluded that the activity of these active mutant enzymes mirrors the results observed in *P. putida* [9,13,25,53].

In addition to L-histidine, *Gk*HAL was tested with various homologous substrates to L-histidine to study its promiscuity or substrate scope. All enzymes that were active with the natural substrate could also react with L-histidine methyl ester. However, only the R280K and F325Y mutants were able to react with L-histidinamide. Previous reports indicate that L-histidine methyl ester serves as a substrate for *Ppu*HAL [20] and acts as an inhibitor of *Achromobacter liquidum* HAL [41]. Moreover, substrates such as 4-nitro L-histidine [19] and Nτ-methylhistidine [21] have also been found to react with HAL. To the best of our knowledge, this is the first report demonstrating that HAL mutants of the active center are active with a substrate other than L-histidine. While ammonia-lyase enzymes are notably characterized by their strong L-enantioselectivity, there is increasing interest in their potential applications for kinetic resolution and deracemization of amino acids [4,31,32]. A key challenge in enhancing the industrial applicability of HAL is to widen its limited substrate range. The site-active mutants under study were engineered to assess their role in hydrolyzing L-histidine as a preferred substrate and to determine if these modifications could expand the substrate scope. Our results show that, despite alterations in its active site, HAL generally retains a restricted substrate scope.

The *K*_m_ value of L-histidine for *Gk*HAL is comparable to those of the closely related *Ppu*HAL (3.9 mM) [25] and *Pf*HAL (2.7 mM) [27]. *Pseudomonas putida* HAL with mutations similar to those studied here also displayed a *K*_m_ range comparable to the values we obtained [9,13,24]. Reactions with other substrates show *K*_m_ values that are somewhat comparable to those for L-histidine across all enzymes, suggesting that the binding to these homologous substrates is similar in each case.

Regardless of the substrate, the wild-type enzyme exhibits significantly higher turnover values, indicating superior catalytic activity compared to the mutant enzymes. Dramatic losses of activity were observed in the R280K and F325Y mutated enzymes, with these losses varying depending on the substrate. Overall, the catalytic efficiencies of the mutant enzymes were found to be lower than those of the wild-type enzymes. The catalytic efficiencies of the Q274N mutant are one to two orders lower due to high *K*_m_ values, which implies an added difficulty in enzyme–substrate binding. This could be because this residue plays a role in maintaining the correct geometry of R280 and Tyr52, as previously suggested for *Ppu*HAL [9,25]. On the other hand, the F325Y mutation showed the opposite behavior. Although its *K*_m_ values are similar to the wild-type HAL, the low catalytic activity resulted in an efficiency rate comparable to the Q274N mutation, at least with the natural substrate. In *Ppu*HAL, the impact of mutating this phenylalanine residue to alanine has shown differential behavior, with activity decreasing by 100 to 2200-fold [25,53]. Moreover, the F329G mutant was inactive with L-histidine due to its failure to form the MIO group [53]. Nonetheless, it is clear that these findings collectively underscore the importance of the phenyl ring in HAL enzyme catalysis. The R280K mutation shows the lowest catalytic function, with its efficiency falling to two orders of magnitude below that of the wild-type enzyme. Surprisingly, the *k*_cat_ value for R280K *Gk*HAL with L-histidinamide is nearly 2.5 times higher than that for the native substrate. This indicates that the mutation allows the enzyme to be more effective with the homologous substrate than with L-histidine. Interestingly, new pathways emerged, as both the R280K and F325Y mutations enabled the enzyme to catalyze the reaction with L-histidinamide, a substrate with which the wild-type *Gk*HAL is inactive.

### 3.3. The Kinetics of Thermal Inactivation of GkHAL Mutants: Relationship with Thermostability

The maximum activity of *Gk*HAL was observed at 85 °C, aligning with the thermophilic nature of its gene donor organism. This high-temperature performance underscores the importance of studying its thermostability and evaluating whether mutations can enhance or diminish this characteristic. The efficiency of the enzymes over time is represented by the inactivation rate constant (*k*_d_) and half-life times (t_1/2_). At lower temperatures, the wild-type *Gk*HAL exhibited the greatest stability over time, while the F325Y mutant enzyme showed superior kinetic stability at higher temperatures. Half-life times found for PAL from *Anabeana variabilis* [54] and *Lactuca sativa* L. [55] at temperatures of 67 and 60 °C were 8 h and 2.8 h, respectively. In comparison, *Gk*HAL seems to better maintain its stability at high temperatures than other aromatic ammonia-lyase enzymes.

The linear behavior of the thermal inactivation plots suggests that HAL inactivation occurs through a mechanism solely influenced by temperature [56,57]. The activation energy for the process resulted in similar range energies but was significantly lower for Q274N and F325Y, indicating easier inactivation consistent with their shorter half-life times. High E_a_ for *Gk*HAL enzymes indicates significant stability at elevated temperatures, consistent with their thermophilic nature [36,58]. Thermodynamic parameters for the enzyme inactivation process were derived from the activation energy. ΔG* values for *Gk*HAL enzymes ranged from 26.04 and 27.68 kcal mol^−1^, which are similar to those reported for other thermophilic enzymes, such as α-amylases from *Bacillus amyloliquefaciens* (22.90 kcal mol^−1^) [42] or β-xylosidase from *Geobacillus stearothermophilus* (27.60 kcal mol^−1^) [43]. However, ΔH* and ΔS* values displayed a higher difference between the wild-type enzyme and the mutated ones. Protein inactivation is an endothermic denaturalization process, as energy is required to alter its structure. ΔH* is a measure of the number of bonds broken during inactivation [59]. ΔH* values for the R280K mutation and the wild-type were higher compared to those for the Q274N and F325Y mutations, suggesting that the latter two mutant enzymes have lower heat resistance. Entropy generally augments with the denaturalization of the protein. ΔS* values are known to provide important insights into solvation levels, protein compactness [59], and aggregation [60]. The positive ΔS* values observed for wild-type *Gk*HAL and the mutants Q274N and R280K indicate an increase in disorder or randomness within these enzymes or the surrounding solvent system during denaturation [45]. In contrast, the negative ΔS* values observed for the F325Y mutant suggest that the partially unfolded state is more ordered compared to the native state structure and may also point to the occurrence of an aggregation process [60]. Finally, the enthalpy and entropy values for wild-type *Gk*HAL are lower than those of other thermophilic enzymes, such as α-amylase [42] and β-xylosidase [43], which may indicate slight differences in the stability mechanisms among these enzymes.

### 3.4. Conformational Stability and Irreversible Thermal Denaturalization

Far-UV CD spectra were employed to assess the secondary structure of *Gk*HAL and to monitor any structural changes and correct folding in its active site mutants [61]. *Gk*HAL enzymes showed secondary structures with α-helices accounting for a significant portion, ranging from 35% to 55% of the total. A previous study demonstrated that *Ppu*HAL exhibits a helical conformation at a higher percentage of 75% [62]. The opposite case can be found in β-sheets, appearing at low percentages for *Gk*HAL and at 0% for *P. putida* [62]. Random conformation was also found to be more present in *G. kaustophilus*. Since it is possible to determine the secondary structure from a PDB structure, we used the BestSel server to analyze the crystal structure (PDB 1B8F) reported for *Ppu*HAL [63]. The analysis revealed an α-helix content of 55%, a very low β-sheet content of 5.7%, and 39.2% turns and other structures (Figure S11). These estimations are consistent with the results obtained for wild-type *Gk*HAL.

The effect of temperature on *Gk*HAL and its mutants, initially investigated through activity measurements, was further assessed by evaluating thermal denaturation using CD spectroscopy. All four proteins displayed sigmoidal curves, indicating that they have well-folded structures. The thermal denaturation of *Gk*HAL and its mutants was found to be irreversible, which precluded the estimation of the thermodynamic parameters governing thermal unfolding [43,47]. Despite this limitation, we were able to determine the apparent melting temperature. This approach aligns with methods used for other proteins that exhibit irreversible thermal denaturation, allowing us to gain insights into their thermal stability. The high *T*_m(app)_ value for wild-type *Gk*HAL confirms its significant thermostability, which is consistent with the kinetic inactivation results obtained. Only the F325Y mutation resulted in a significantly lower melting temperature, suggesting the importance of this residue for proper folding. In *Ppu*HAL, the analogous residue (F329) is proposed to stabilize the σ-complex-like conformation [9,13,25]. These findings are consistent with the thermodynamic data on enzymatic thermal inactivation, which demonstrate that the F325Y mutant requires the lowest energy for denaturation. As a result, the F325Y mutant appears to be the most unstable of the three mutants at elevated temperatures.

## 4. Materials and Methods

### 4.1. Materials

All chemicals were of analytical grade and were used without further purification. Restriction enzymes and T4 DNA ligase were purchased from Roche Diagnostic S.L. (Indianapolis, IN, USA). Kapa-Hifi polymerase was from KapaBiosistems (Cultek S.L.U., San Fernando de Henares, Spain). Primers were from Integrated DNA Technologies, IDT (Coralville, IA, USA). TALON TM metal affinity resin was purchased from Clontech Laboratories, Inc. (Takara Scientific Chemical, Waltham, MA, USA). L- and D-histidine were from Alfa Aesar GmbH & Co. K.G. (Thermo Fischer Scientific, Waltham, MA, USA), and *trans*-urocanic acid was from Acros Organics (Thermo Fischer Scientific, Waltham, MA, USA). L-histidinamide, 1-methyl-L-histidine, 3-methyl-L-histidine, and 2-mercapto-L-histidine were from Sigma Aldrich (St. Louis, MO, USA), benzyl-L-histidine and β-alanyl-L-histidine were from TCI Europe (Zwijndrecht, Belgium), L-histidine methyl ester was purchased from Acros Organics (Thermo Fischer Scientific, Waltham, MA, USA), and diamine L-histidine was from Bachem (Bubendorf, Switzerland). Diethanolamine was purchased from Panreac (Castellar del Vallés, Spain), acetic acid, formic acid, monobasic sodium phosphate, and dibasic potassium phosphate were from Merck (Darmstadt, Germany), and sodium carbonate was from Riedel de Haën (Honeywell, Charlotte, NC, USA).

### 4.2. Microbes and Culture Conditions

*G. kaustophilus* CECT4264, acquired from the Spanish Type Culture Collection (CECT), was used as a possible donor of the histidine ammonia-lyase gene (*hutH*). The strain was cultivated at 55 °C for 20 h in Luria–Bertani medium (LB) (1% tryptone, 0.5% yeast extract, 0.5% NaCl, pH 7.2). *E. coli* DH5α was used to clone the gene *hutH*, and *E. coli* BL21 was used to overexpress the recombinant protein.

### 4.3. Cloning of the Histidine Ammonia-Lyase (hutH) Gene

A single-colony isolate of *G. kaustophilus* CECT4264 was chosen for DNA isolation using a boiling procedure [49]. A supernatant aliquot of 5 µL containing the genomic DNA of the strain was used to amplify the gene encoding for putative *hutH* by PCR. The primers used to amplify the gene from *G. kaustophilus* CECT4264 (*gkhutH*) were designed based on GenBank sequence accession number NC_006510.1. These were GkHAL5 (5′-GGAATT*CCATATG*ATCGTATTGA-CCGGGC-3′) and GkHAL3 (5′-CG*GGATCC*AGCTTTCGCTTCATCGCGG-3′), including *Nde* I and *Bam*H I restriction sites, respectively (in italics in the primer sequence).

The PCR product was purified from an agarose gel using the E.Z.N.A. Gel Extraction Kit (Omega Bio-Tek, Norcross, GA, USA), treated with *Nde* I and *Bam*H I enzymes (New England Biolabs, Ipswich, MA, USA), and then ligated into the pDANI3 plasmid, which was cut with the same enzymes to create the plasmid pJMC93. The plasmid pDANI3 was made in our laboratory [64] and derived from the rhamnose-inducible expression vector, including a C-terminal His6-tag (pJOE4036.1) [65] and including the TEV (Tobacco Etch Virus) recognition site, ENLYFQS [66]. The resulting construct allows the production of recombinant *Gk*HAL fused at the C terminus to a polyhistidine tag (His6 tag), allowing its removal by TEV treatment. The cloned sequence was confirmed by sequencing using an Applied Biosystems 3500 Series Genetic Analyzer (Thermo Fisher Scientific, Waltham, MA, USA) at the Nucleic Acids Analysis Service of the University of Almeria. The sequence was then aligned and compared as previously described [49].

Mutagenesis of *Gk*HAL was performed using the QuikChange II Site-directed mutagenesis kit from Stratagene (San Diego, CA, USA) following the manufacturer’s protocol, using the plasmid pJMC93 as the template and the primers described in Table S1. Mutations (Tyr52Phe, His82Leu, Asn194Asp, Gln274Asn, Tyr277Phe, Arg280Lys, Phe325Tyr, and Glu411Asp) were also confirmed by DNA sequencing.

### 4.4. Expression and Purification of the GkHAL Enzyme and Mutants

The protocol was similar to that described in [61]. *E. coli* BL21/pJMC93 and the mutated strains were grown in an LB medium supplemented with ampicillin. For induction and expression of the genes, L-rhamnose was added to the culture and incubated at 30 °C overnight. The cells were collected by centrifugation (Beckman J-26 XPI centrifuge) (6500× *g*, 4 °C, 25 min) (Beckman Coulter, Brea, CA, USA) and stored at −20 °C until use.

The cell wall disruption was performed by sonication as described before [47] after resuspension in the wash buffer. The enzyme purification was performed by metal affinity chromatography using cobalt resin [38]. Protein purity was determined at different stages of the purification by SDS-PAGE electrophoresis. Additional gel filtration chromatography was carried out to eliminate any DNA or protein co-eluting with the protein of interest [61]. The purified enzymes were concentrated using Vivaspin concentrators (Sartorius, Gotinga, Germany), dialyzed against 100 mM diethanolamine (DEA) buffer, pH 8.5, and stored at 4 °C. Protein concentrations were determined from the absorbance of the extinction coefficient of tyrosine residues [67].

### 4.5. Molecular Mass Analysis

Size-exclusion chromatography–high-pressure liquid chromatography (SEC-HPLC) analysis was conducted to determine the molecular mass of both the wild-type and mutated *Gk*HAL enzymes in their native states. The HPLC System (Breeze HPLC System, Waters, Barcelona, Spain) used a Biosep-SEC-S2000 column (Phenomenex, Madrid, Spain) that was calibrated using a non-denatured protein molecular weight marker kit (Bio-Rad, Madrid, Spain) [38,47]. The molecular mass of the monomeric form of both the wild-type and mutated *Gk*HAL enzymes was determined using sodium dodecyl-sulfate polyacrylamide gel electrophoresis (SDS-PAGE) with a low molecular weight marker kit (Cytiva, Marlborough, MA, USA).

### 4.6. Enzyme Assay and Protein Determination

The *Gk*HAL activity assay was based on the transformation of L-histidine to give *trans*-urocanic acid. The activity was measured with the spectrophotometer ThermoScientific NanoDrop 2000 (Waltham, MA, USA) at 280 nm. Standard enzymatic assay was carried out by adapting the method described in [68] with the purified *Gk*HAL and mutated enzymes (at final concentrations from 0.4 to 4.3 µM), along with L-histidine as the substrate (1 mM) dissolved in 100 mM DEA buffer (pH 8.5), in a reaction volume of 500 µL at 65 °C. The protein was added to 485 µL of DEA buffer, and the reaction was started with the addition of 5 µL of a 0.1 M L-histidine solution. The activities of both wild-type and mutant enzymes were assessed. More active enzymes were measured at intervals of 1 to 5 min, while less active ones were measured at intervals of 5 to 30 min. The activity was determined utilizing a molar extinction coefficient for *trans*-urocanic acid of 18,800 M^−1^ cm^−1^, as outlined in [69].

The optimal pH of purified *Gk*HAL wild-type and its mutants was determined by conducting the standard assay in the appropriate buffers ranging from 1.92 to 11.9: phosphoric buffer (pH 2–3), formic buffer (pH 3–4), acetate buffer (pH 4–5.5), phosphate buffer (pH 6–7.5), Tris-HCl buffer (pH 8 and pH 9), carbonate buffer (pH 9.5–11), and sodium phosphate buffer (pH 11.5–12). The effect of the temperature on the *Gk*HAL wild-type and its mutant forms was studied by performing the standard enzymatic assay within the temperature range of 4 to 99 °C. Thermal stability of *Gk*HAL wild-type was determined after 15 min of preincubation at different temperatures from 4 to 90 °C in 100 mM DEA buffer pH 8.5, followed by the standard activity assay.

*Gk*HAL activity on other substrates was studied with the standard assay, only varying L-histidine to a different reactive. In addition to the proteinogenic amino acids, *Gk*HAL activity was tested on D-histidine, L-histidinamide, L-histidine methyl ester, bencyl-L-histidine, β-alanyl-l-histidine, 1-methyl-L-histidine, 3-methyl-L-histidine, β-mercapto-L-histidine, and diamine-L-histidine, with a substrate concentration in reaction of 1 mM.

Determination of *K*_m_ and *V*_max_ for L-histidine, L-histidine methyl ester, and L-histidinamide was performed by varying substrate concentrations from 0.25 to 50 mM. Kinetic parameters (*K*_m_, *V*_max_) were calculated with non-linear square hyperbole plots following the Michaelis–Menten equation [56]. The fitting was made using SigmaPlot^®^ (version 12.0, Systat Software, Inc., San Jose, CA, USA). The turnover number (*k*_cat_) was calculated by dividing *V*_max_ by the enzyme concentration. The catalytic efficiency was determined through the ratio of *k*_cat_ to *K*_m_ (*k*_cat_/*K*_m_).

Thermal inactivation kinetics were determined by performing the standard assay with previous incubations of the enzymes at 65, 70, 75, and 80 °C. Pre-incubation times vary from 5 min to 60 min for higher temperatures and from 1 to 24 h for lower temperatures. The inactivation rate constant (*k*_d_) of the enzymes was determined using relative residual activities fitted to Arrhenius plots. The half-life value (t_1/2_) of the enzymes at a particular temperature was then determined from each *k*_d_. Activation parameters, including activation energy for denaturation and changes in enthalpy, free energy, and entropy for each incubation temperature, were determined as outlined in references [42,44,45,57].

### 4.7. Circular Dichroism

Circular dichroism spectra were recorded using a Jasco J815 spectropolarimeter equipped with a thermostated cell holder and interfaced with a Peltier accessory.

Far-UV spectra were performed to assess the secondary structure of wild-type and mutant *Gk*HAL enzymes. The protein concentration was 4 μM (in protomer units) in 50 mM DEA buffer, pH 8.5. CD spectra were acquired at a scan speed of 50 nm/min with a response time of 4 s at 25 °C and averaged over six scans spanning from 190 to 260 nm. Spectra were corrected by subtracting the baseline scan (buffer). The secondary protein structure was decomposed and analyzed using the k2d algorithm online on the DichroWeb site [46].

Thermal denaturalization of *Gk*HAL and its mutants was performed with a total protein concentration of 4 μM in an interval of temperatures of 25 to 95 °C with an increase of 60 °C per hour. Data were collected every 0.2 °C at 222 nm with an 8 s response and 1 nm of bandwidth. Precipitation occurred in all cases upon heating.

The thermal transitions of wild-type and *Gk*HAL mutants were analyzed using a two-state model [47]. The spectral parameters were directly fitted to the following equation through non-linear least squares analysis:

(1) $$S_{\mathrm{obs}}=\frac{S_{N}+S_{U\;}\exp\left(-\frac{\Delta H_{\mathrm{VH}}}{R}\left(\frac{1}{T}-\frac{1}{T_{m}}\right)\right)}{1+\exp\left(-\frac{\Delta H_{\mathrm{VH}}}{R}\left(\frac{1}{T}-\frac{1}{T_{m}}\right)\right)}$$

where *S*_obs_ represents the ellipticity at 222 nm, and *S*_N_ = A_N_ + B_N_*T* and *S*_U_ = A_U_ + B_U_*T* refer to the linear dependence of the native (N) and unfolded (U) states, which have the slopes B_N_ and B_U_ and the intercepts A_N_ and A_U_, respectively. Δ*H*_VH_ corresponds to the apparent change in van’t Hoff enthalpy, *R* is the universal gas constant, *T* is the temperature in Kelvin, and *T_m_* is the transition midpoint at which 50% of the protein is unfolded. Data fitting was carried out with Kaleidagraph (https://www.synergy.com/).

### 4.8. Nucleotide Sequence Accession Number

The nucleotide sequence of *Gk*HAL has been deposited in the GenBank database under accession No. PQ065632.

## 5. Conclusions

This study successfully characterized a novel histidine ammonia-lyase from *Geobacillus kaustophilus*, highlighting its high thermal stability and catalytic efficiency. The mutational analysis provided critical insights into the structure–function relationships within the enzyme, revealing essential residues for catalytic activity and identifying mutations that expand substrate specificity. These results not only contribute to our understanding of HAL enzymes but also underscore their potential for industrial applications, particularly in environments demanding high thermal resilience. Future work could investigate the immobilization of these enzymes to further enhance their thermostability, with the aim of evaluating their potential use as industrial biocatalysts.

## Figures and Tables

**Table 1:** Kinetic constants and velocity rates for *Gk*HAL enzymes.

Enzyme	Substrate	*K*_m_	*k*_cat_	*V*_max_	*k*_cat_/*K*_m_ (rel. Decrease)
		mM	s^−1^	×10^−7^ mol min^−1^	mM^−1^ s^−1^
WT *Gk*HAL	L-histidine	4.83	12.239	4.243	2.5367 (1)
	L-histidine methyl ester	8.77	13.243	4.591	1.5101 (1)
Q274N *Gk*HAL	L-histidine	12.58	1.925	0.628	0.1530 (17)
	L-histidine methyl ester	47.21	2.428	0.793	0.0514 (29)
R280K *Gk*HAL	L-histidine	7.99	0.096	0.231	0.0120 (211)
	L-histidine methyl ester	3.43	0.090	0.021	0.0262 (58)
	L-histidinamide	5.39	0.220	0.052	0.0408
F325Y *Gk*HAL	L-histidine	3.38	0.547	0.273	0.1617 (16)
	L-histidine methyl ester	22.98	0.454	0.227	0.0197 (77)
	L-histidinamide	5.40	0.008	0.004	0.0015

**Table 2:** Thermodynamic parameters for thermal inactivation of *Gk*HAL enzymes.

Incubation Temperature	Half-Life	Inactivation Rate Constant *k*_d_	ΔG*	ΔH*	ΔS*
K	min	min^−1^	kcal mol^−1^	kcal mol^−1^	kcal K^−1^ mol^−1^
Wild-type *Gk*HAL (E_a_ = 49.42 kcal mol^−1^)					
353	34	0.0206	26.38	48.72	0.063
348	224	0.0031	27.30	48.73	0.062
343	301	0.0023	27.10	48.74	0.063
338	990	0.0007	27.50	48.75	0.063
Q274N *Gk*HAL (E_a_ = 35.64 kcal mol^−1^)					
353	71	0.0097	26.91	34.93	0.023
348	385	0.0018	27.68	34.94	0.021
343	408	0.0017	27.31	34.95	0.022
338	866	0.0008	27.41	34.96	0.022
R280K *Gk*HAL (E_a_ = 44.41 kcal mol^−1^)					
353	21	0.0335	26.04	43.71	0.050
348	187	0.0037	27.18	43.72	0.048
343	533	0.0013	27.49	43.73	0.047
338	770	0.0009	27.33	43.74	0.049
F325Y *Gk*HAL (E_a_ = 25.11 kcal mol^−1^)					
353	88	0.0079	27.05	24.42	-0.007
348	112	0.0062	26.82	24.43	-0.007
343	182	0.0038	26.76	24.44	-0.007
338	433	0.0016	26.94	24.45	-0.007

**Table 3:** Estimated secondary structure composition (%) of wild-type *Gk*HAL and its mutants Q274, R280K, and F325Y determined using a k2d program, and their apparent melting values.

Enzyme	α-Helices (%)	β-Sheets (%)	Random Coil (%)	*T*_m(app)_^ a^ (K)	∆*T*_m(app)_^b^ (K)
WT *Gk*HAL	47	15	38	357.36 ± 0.06	-
Q274N *Gk*HAL	39	20	41	355.20 ± 0.28	−2.16
R280K *Gk*HAL	55	9	36	358.80 ± 0.04	1.40
F325Y *Gk*HAL	35	18	47	351.85 ± 0.14	−5.51

^a^ *T*_m(app)_ is the temperature at which 50% of the protein is unfolded. ^b^ Change in *T*_m(app)_ relative to the wild-type protein.

## Data Availability

The data presented in this article are available from the authors upon request.

## Supplementary Materials

The supporting information can be downloaded at: https://www.mdpi.com/article/10.3390/ijms251810163/s1.
