# Supplementary material for: Exploring the Kinetics and Thermodynamics of a Novel Histidine Ammonia-Lyase from Geobacillus kaustophilus
Source: Int J Mol Sci. 2024 Sep 21;25(18):10163. doi: 10.3390/ijms251810163 (PMC11432326; doi:10.3390/ijms251810163)
Supplement: Supplementary file 1 [file ijms-25-10163-s001.zip › ijms-3209019-supplementary.pdf]

Exploring the Kinetics and Thermodynamics of a Novel Histidine Ammonia-Lyase from *Geobacillus kaustophilus*

Francisco Manuel Salas-Garrucho [1], Alba Carrillo-Moreno [1], Lellys M. Contreras [1*], Felipe Rodríguez-Vico [1,2], Josefa María Clemente-Jiménez [1,2] and Francisco Javier Las Heras-Vázquez [1,2*]

[1]Departamento de Química y Física, Universidad de Almería, 04120 Almería, Spain.
[2]Campus de Excelencia Internacional Agroalimentario ceiA3, University of Almería, 04120 Almería, Spain

*Correspondence authors:

Lellys M. Contreras. Departamento de Química y Física, Universidad de Almería, 04120 Almería, Spain. email: mariela@ual.es.

Francisco Javier Las Heras-Vázquez. Departamento de Química y Física, Universidad de Almería, 04120 Almería, Spain. email: fjheras@ual.es.

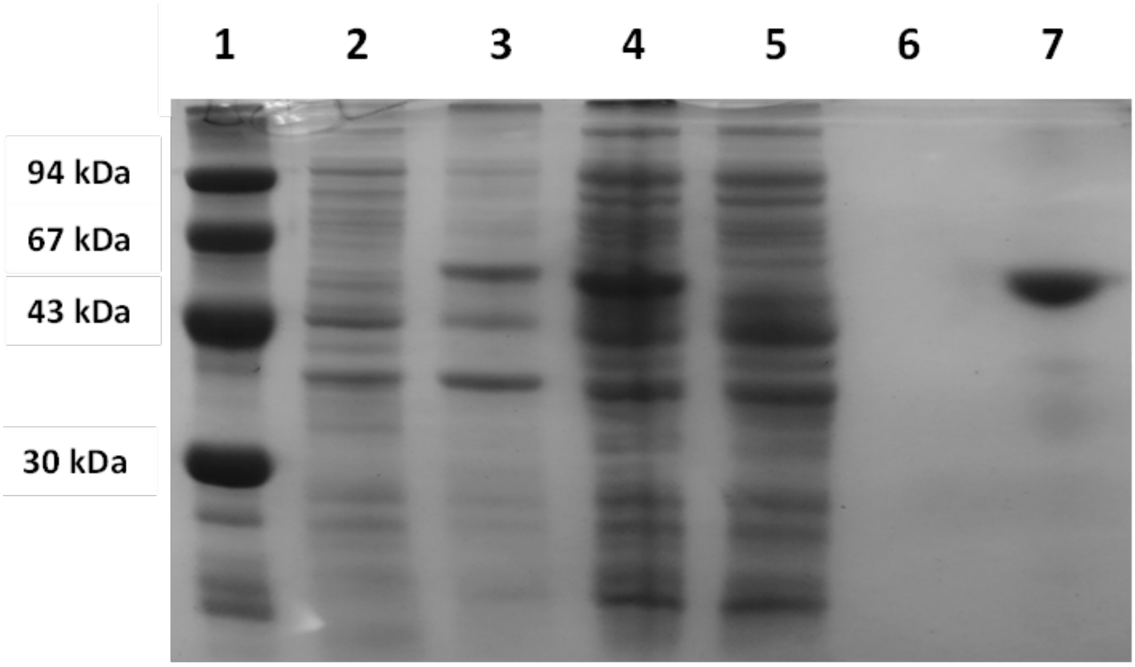

Figure S1. SDS-PAGE analysis of purified *Gk*HAL Y52F. Lane 1: molecular mass marker (30-94 kDa); lane 2: non-inducted precipitate; lane 3: inducted precipitate; lane 4: inducted supernatant; lane 5: eluent; lane 6: wash buffer; lane 7: concentrated enzyme.

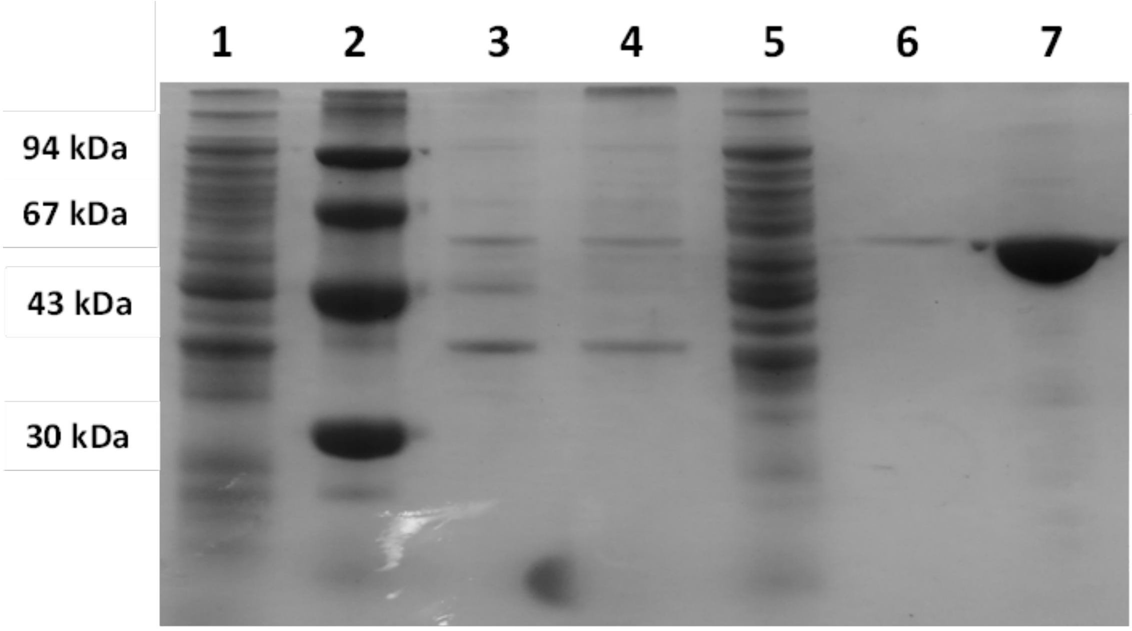

Figure S2. SDS-PAGE analysis of purified *Gk*HAL H82L. Lane 1: non-inducted precipitate; lane 2: molecular mass marker (30-94 kDa); lane 3: inducted precipitate; lane 4: inducted supernatant; lane 5: eluent; lane 6: wash buffer; lane 7: concentrated enzyme.

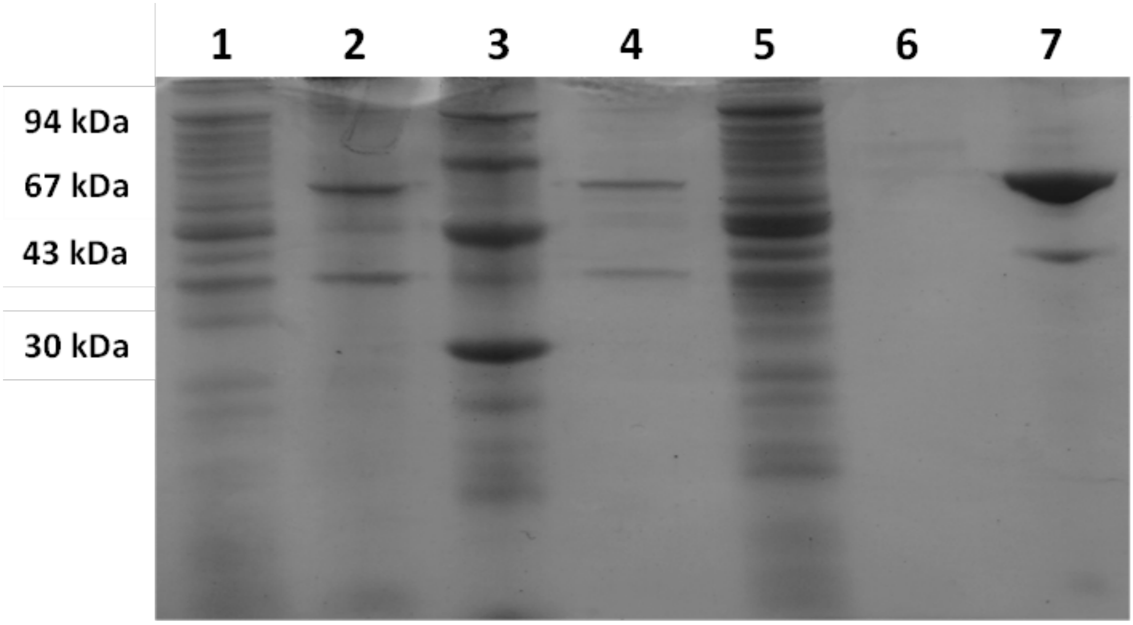

Figure S3. SDS-PAGE analysis of purified *Gk*HAL N194A. Lane 1: non-inducted precipitate; lane 2: inducted precipitate; lane 3: molecular mass marker (30-94 kDa); lane 4: inducted supernatant; lane 5: eluent; lane 6: wash buffer; lane 7: concentrated enzyme.

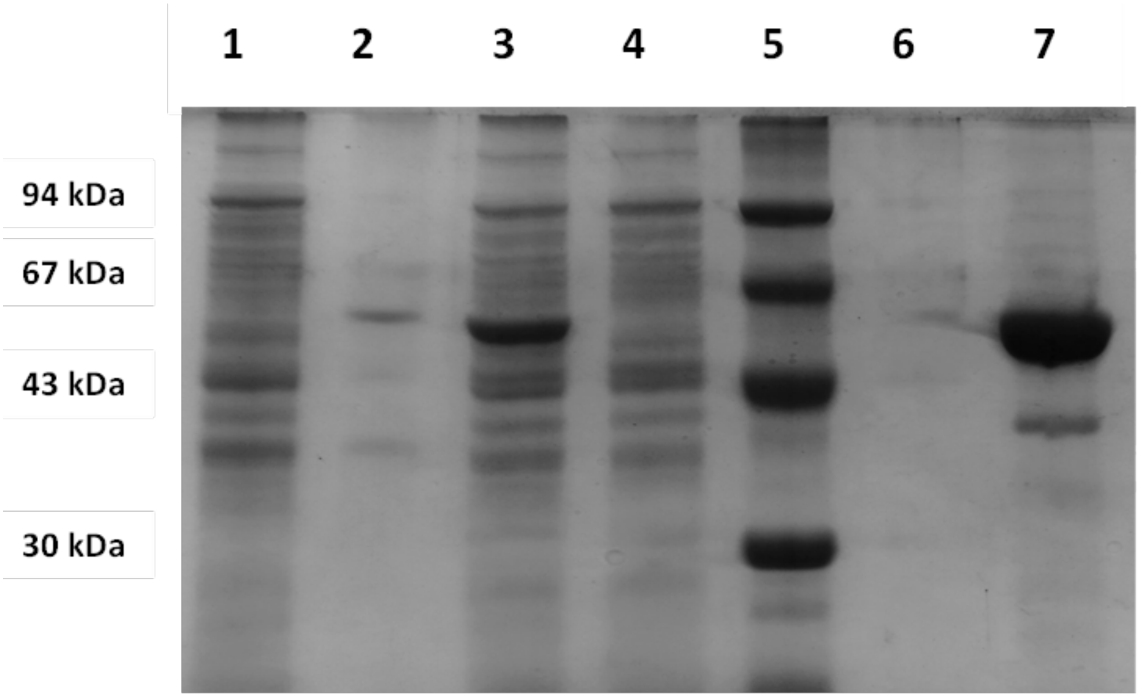

Figure S4. SDS-PAGE analysis of purified *Gk*HAL Q274N. Lane 1: non-inducted precipitate; lane 2: inducted precipitate; lane 3: inducted supernatant; lane 4: eluent; lane 5: molecular mass marker (30-94 kDa); lane 6: wash buffer; lane 7: concentrated enzyme.

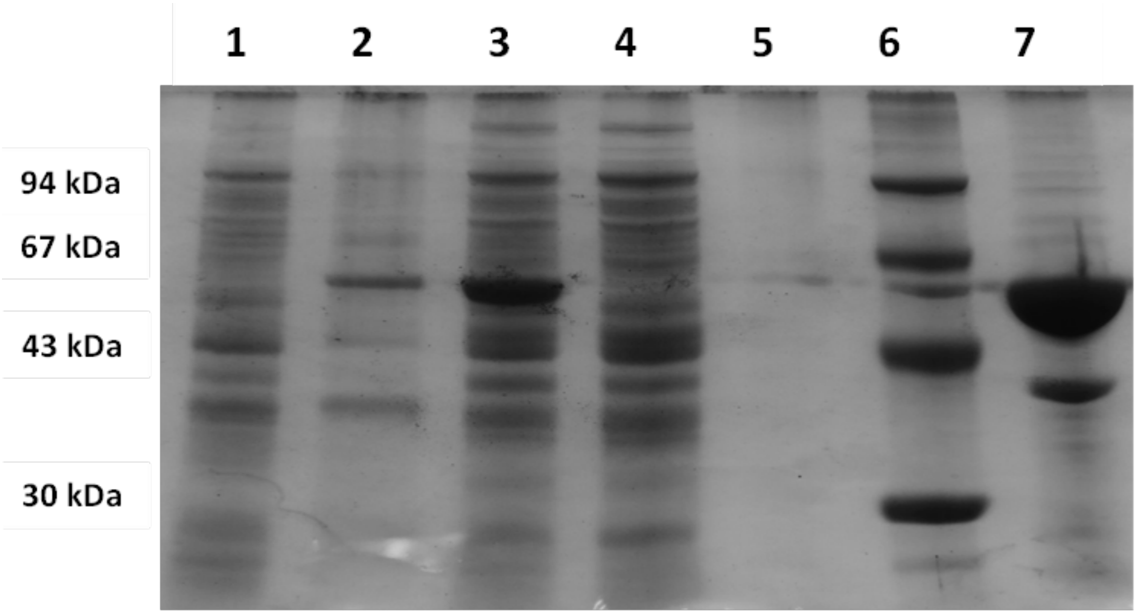

Figure S5. SDS-PAGE analysis of purified *Gk*HAL R280K. Lane 1: non-inducted precipitate; lane 2: inducted precipitate; lane 3: inducted supernatant; lane 4: eluent; lane 5: wash buffer; lane 6: molecular mass marker (30-94 kDa); lane 7: concentrated enzyme.

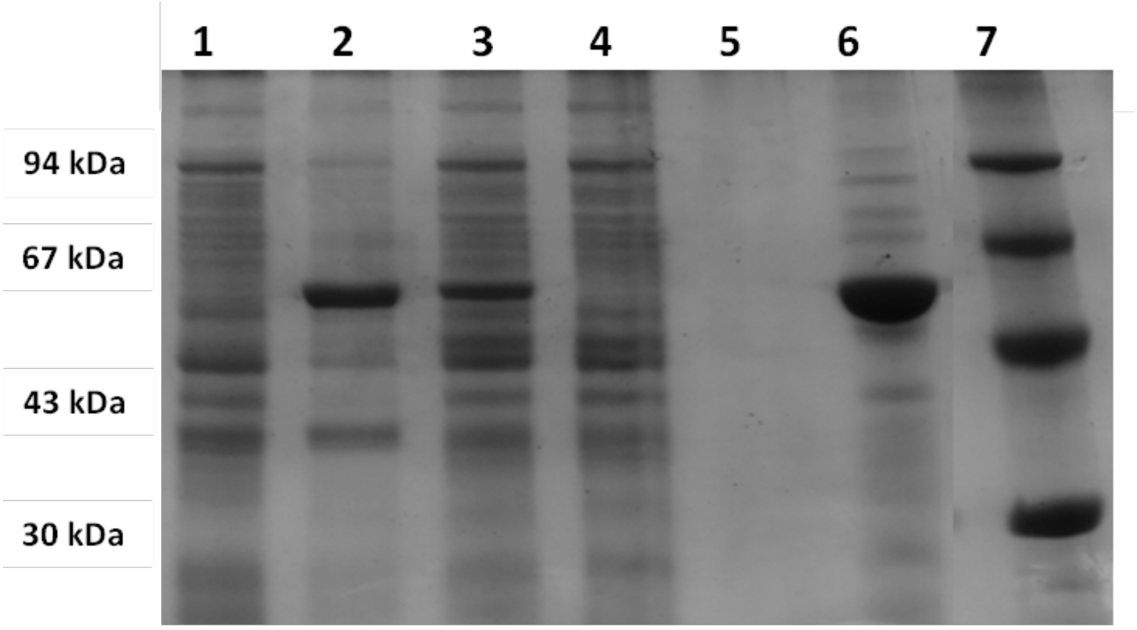

Figure S6. SDS-PAGE analysis of purified *Gk*HAL F325Y. Lane 1: non-inducted precipitate; lane 2: inducted precipitate; lane 3: inducted supernatant; lane 4: eluent; lane 5: wash buffer; lane 6: concentrated enzyme; lane 7: molecular mass marker (30-94 kDa).

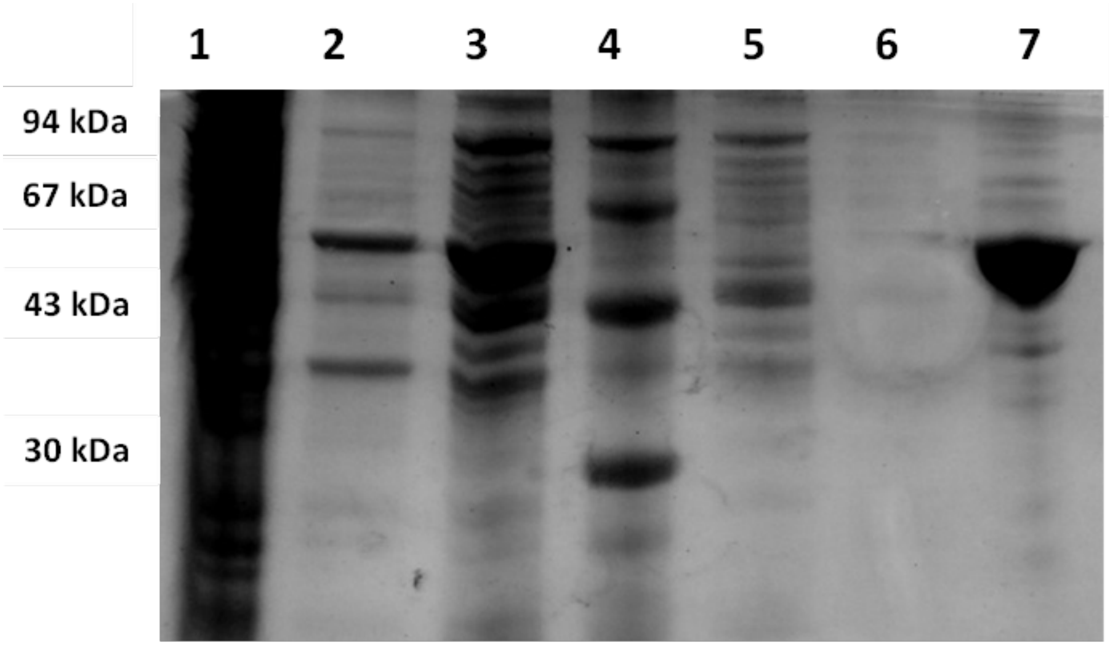

Figure S7. SDS-PAGE analysis of purified *Gk*HAL E411D. Lane 1: non-inducted precipitate; lane 2: inducted precipitate; lane 3: inducted supernatant; lane 4: molecular mass marker (30-94 kDa); lane 5: eluent; lane 6: wash buffer; lane 7: concentrated enzyme.

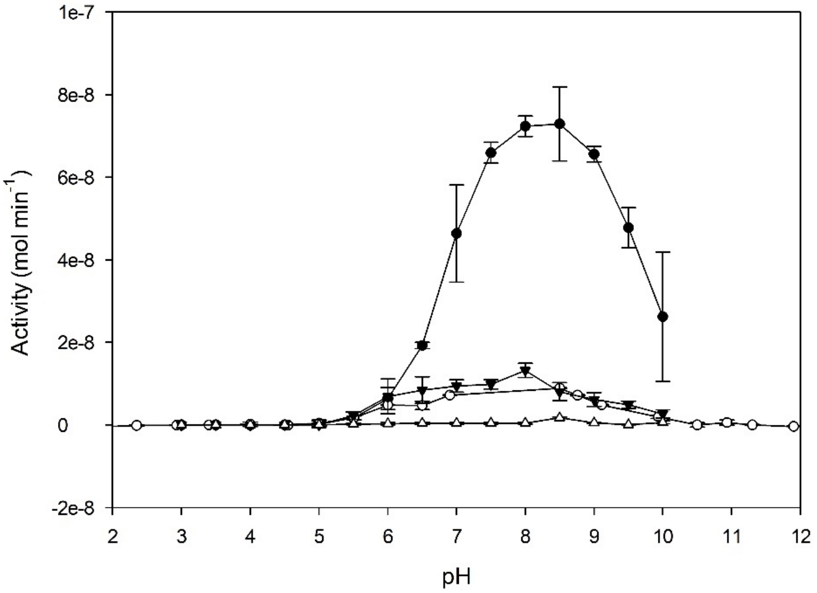

Figure S8. Effect of pH on the activity of *Gk*HAL proteins using L-histidine as substrate. Wild-type *Gk*HAL (close circles), Q274N *Gk*HAL (open circles), R280K *Gk*HAL (close inverted triangles) and F325Y *Gk*HAL (open triangles).

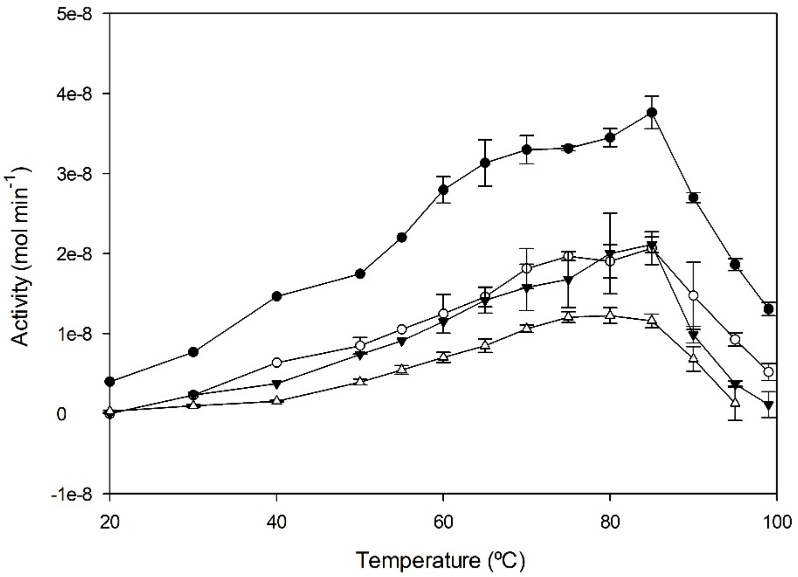

Figure S9. Effect of temperature in the activity of *Gk*HAL proteins at pH 8.5 in DAE 0.1 M and L-histidine as substrate. Wild-type *Gk*HAL (close circles), Q274N *Gk*HAL (open circles), R280K *Gk*HAL (close inverted triangles) and F325Y *Gk*HAL (open triangles).

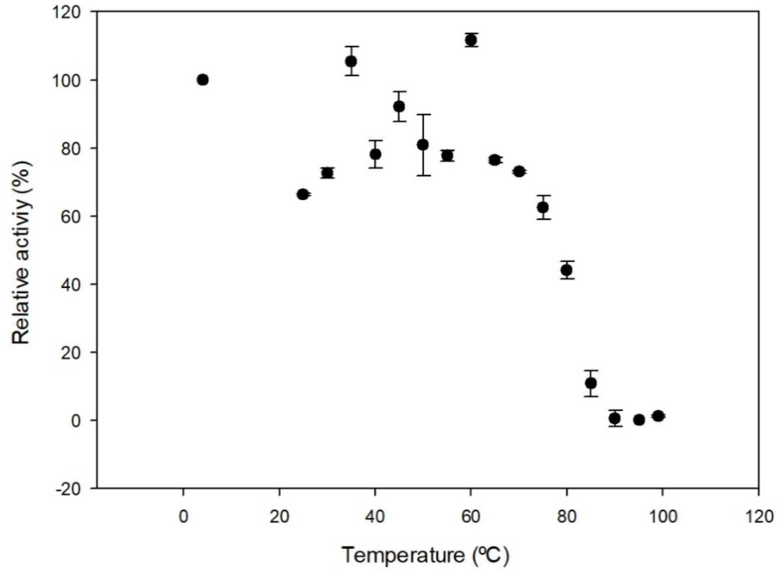

Figure S10. Remaining relative activity of wild-type *Gk*HAL after 15 minutes of pre-incubation at the indicated temperatures. The remaining activity was assayed following the standard activity assay.

References: Micsonai et al. Nucleic Acids Res. 46:W315-22 (2018), Micsonai et al. PNAS 11:E3095-103 (2015)

PDB ID: **1B8F** Entire structure Length: **507**

Secondary structure composition

DSSP [a]	%
3-10-helix	**4.1**
Alpha helix	**55.0**
Pi helix	**0.0**
Turn	**10.8**
Strand	**5.7**
Bridge	**0.4**
Bend	**5.1**
Loop + missing	**18.7**

BeStSel	%
Helix 1	**39.2**
Helix 2	**15.8**
Antiparallel 1	**0.4**
Antiparallel 2	**0.4**
Antiparallel 3	**3.7**
Parallel	**1.2**
Turn	**10.8**
Others	**28.4**

SELCON [b]	%
Helix 1	**40.4**
Helix 2	**18.7**
Strand 1	**2.2**
Strand 2	**3.5**
Turn	**13.0**
Others	**22.1**

CATH Info [c]

CATH info is provided on the basis of chains. Please, select chains below.

Further references:

[a] : Dictionary of protein secondary structure: pattern recognition of hydrogen-bonded and geometrical features.
Kabsch W., Sander C.
Biopolymers. 1983 Dec; 22(12):2577-2637. PMID: 6667333

[b] : Estimation of the number of alpha-helical and beta-strand segments in proteins using circular dichroism spectroscopy.
Sreerama N., Venyaminov SY., Woody RW.
Protein Sci. 1999 Feb;8(2):370-380. PMID: 10048330

[c] : CATH: A Hierarchic Classification of Protein Domain Structures.
Orengo CA., Michie AD., Jones S., Jones DT., Swindells MB., Thornton JM.
Structure. 1997 Aug 15;5(8):1093-108. PMID: 9309224

Figure S11. Secondary structure composition of the crystalline structure of *Ppu*HAL (PDB 1B8F) analyzed using the BeStSel web server.

Table S1. PCR primers for site-directed mutagenesis of the gene *gkhutH* cloned in pJMC93. The native amino acid and the mutation are shown in brackets in the second column. The nucleotides changed for the mutation are highlighted in bold in the primer sequence.

Primer name	Mutation	Sequence
GKHALY52F5	Y52F (TAT/TTT)	5´-TGGACGGACGATCT**T**TGGCGTGAATACGGG-3´
GKHALY52F3	Y52F (TAT/TTT)	5´-CCCGTATTCACGCCA**A**AGATCGTCCGTCCA-3´
GKHALH82L5	H82L (CAC/CTC)	5´-TCTTCTTCGCTCGC**T**CGCTTGTGCCGTCGG-3´
GKHALH82L3	H82L (CAC/CTC)	5´-CCGACGGCACAAGCG**A**GCGAGCGAAGAAGA-3´
GKHALN194A5	N194A (AAC/ **GCC**)	5´-GGCTGGCGCTTATT**GC**CGGCACGCAGGTGA-3´
GKHALN194A3	N194A (AAC/ **GCC**)	5´-TCACCTGCGTGCCG**GC**AATAAGCGCCAGCC-3´
GKHALQ274N5	Q274N (CAA/ **AAT**)	5´-GAGCGGCGCGTG**AAT**GATGCGTACTCCAT-3´
GKHALQ274N3	Q274N (CAA/ **AAT**)	5´-ATGGAGTACGCATC**ATT**CACGCGCCGCTC-3´
GKHALY277F5	Y277F (TAC/TTC)	5´-CGTGCAAGATGCGT**T**CTCCATCCGCTGCCT-3´
GKHALY277F3	Y277F (TAC/TTC)	5´-AGGCAGCGGATGGAG**A**ACGCATCTTGCACG-3´
GKHALR280K5	R280K (CGC/**AAG**)	5´-GCGTACTCCATC**AAG**TGCCTCCCGCAAGTG-3´
GKHALR280K3	R280K (CGC/**AAG**)	5´CACTTGCGGGAGGCA**CTT**GATGGAGTACGC-3´
GKHALF325Y5	F325Y (TTT/TAT)	5´-GGCGGCAACT**A**TCATGGCCAACCCGTTGCG-3´
GKHALF325Y3	F325Y (TTT/TAT)	5´-CGCAACGGGTTGGCCATGA**T**AGTTGCCGCC-3´
GKHALE411D5	E411D (GAG/GAT)	5´- TCAGCGAACCAAGA**T**GATCACGTCAGCATG -3´
GKHALE411D3	E411D (GAG/GAT)	5´-CATGCTGACGTGATC**A**TCTTGGTTCGCTGA-3´
